# The Composition and Function of the Rhizosphere Bacterial Community of *Paeonia lactiflora* Varies with the Cultivar

**DOI:** 10.3390/biology12111363

**Published:** 2023-10-24

**Authors:** Liping Yang, Xin Wan, Runyang Zhou, Yingdan Yuan

**Affiliations:** 1 College of Horticulture and Landscape Architecture, Yangzhou University, Yangzhou 225009, China; yzu_yangliping@163.com (L.Y.); ryzhou2004@126.com (R.Z.); 2 Jiangsu Academy of Forestry, Nanjing 211153, China; lkywanxin@163.com; 3 Jiangsu Yangzhou Urban Forest Ecosystem National Observation and Research Station, Yangzhou 225006, China

**Keywords:** *Paeonia lactiflora*, microbiome, rhizosphere soil, bacterial community

## Abstract

**Simple Summary:**

Rhizosphere microbial composition was affected by plant cultivars, soil properties, climate and other conditions. However, the effects of cultivars on the structure and the function of rhizosphere microbial communities have not been reported completely. In this study, the rhizosphere bacterial community composition of eight cultivars of *Paeonia lactiflora* was revealed by high-throughput sequencing technology. The differences and similarities of rhizosphere bacterial community composition and rhizosphere bacterial interaction among different cultivars were analyzed. In addition, cultivation-related microbial resources that can be used for bioremediation, organic degradation and disease resistance have been identified. This research will contribute to the development of rhizosphere microbial resources of *P. lactiflora* and provide guidance for agricultural practice in the future.

**Abstract:**

The composition and diversity of the rhizosphere microbial community maintain the stability of the root microclimate, and several studies have focused on this aspect of rhizosphere microorganisms. However, how these communities vary with cultivars of a species is not completely understood. *Paeonia lactiflora*—a perennial herb species of the family Paeoniaceae—includes a wide variety of cultivars, with rich rhizosphere microbial resources. Hence, we studied the differences in rhizosphere bacterial communities associated with eight *P. lactiflora* cultivars. We noted that Actinobacteria, Proteobacteria, Acidobacteria, Bacteroidetes, Firmicutes, Verrucomicrobia, Planctomycetes and Chloroflexi were the dominant phyla associated with the cultivars. The composition of rhizosphere bacterial community of different cultivars was highly similar at taxonomic levels, but there were slightly differences in the relative abundance. LEfSe analysis showed that the cultivars “Sheng Tao Hua” and “Zi Lou Xian Jin” exhibited the most biomarkers. Differential ASV analysis revealed the maximum difference in ASV abundance between “Lian Tai” and “Zi Hong Zheng Hui”, as well as between “Sheng Tao Hua” and “Tao Hua Fei Xue”, and the maximum similarity between “Duo Ye Zi” and “Xue Feng”. Co-occurrence network analysis revealed that rhizosphere bacteria in most cultivars maintain homeostasis by cooperation, wherein Actinobacteria and Proteobacteria played a vital role. In addition, microbial resources related to cultivars like bioremediation, organic degradation and resistance to diseases are found. This study revealed the structures of the rhizosphere bacterial communities associated with different cultivars of *P. lactiflora* and explored their stress resistance potential, which can be used to guide future agricultural practices.

## 1. Introduction

The rhizosphere refers to the narrow range of soil affected by root secretions, and rhizosphere microorganisms, often called the “second genome of plants”, grow and reproduce in this range [1,2]. They interact closely with plants, promote plant growth and development through various mechanisms, inhibit pathogen activities, and enhance plant resistance to stress conditions [3,4,5,6]. In turn, root exudates play an important role in shaping the plant rhizosphere microbiome [7]. To drive and regulate the diversity and vitality of rhizosphere microorganisms, plant roots actively secrete nutrients into the surroundings to form rhizosphere deposits, which contain signals and attractor molecules that can recruit beneficial microorganisms [2,8]. The innate immune response mediated by rhizosphere microorganisms can resist plant pathogens, and the beneficial strains recruited by microbial communities, along with their characteristics, can enhance the immune function of plant hosts [9]. Kotoky et al. recognized the potential of rhizosphere microorganisms to enhance crop drought resistance, increase yield and optimize soil nutrient cycling, and worked on improving the understanding of plant–microbial interactions [10]. Xu et al. also supported the role of core rhizosphere microorganisms in promoting plant growth and health [11]. These studies suggested that rhizosphere microorganisms are closely related to plants, and it is extremely important to explore and utilize efficient rhizosphere microbial resources to maintain plant growth and development.

The rhizosphere microbial community is influenced by several factors, including plant genotypes, soil properties, climatic conditions and plant nutritional status [12]. Previous studies have revealed that the microbial community structures of the rhizospheres of different cultivars of the same species are different. The rhizosphere microbiomes of wheat cultivars Jimai22 and Xiaoyan22 exhibited different abundances of *Actinobacteria*, *Bacteroidetes*, *Firmicutes*, *Acidobacteria* and *Proteobacteria* [13]. The rhizosphere microbial communities of tobacco cultivars Yunyan87 and Fandi3 were also significantly different [14]. Similarly, different tea cultivars exhibit varying degrees of impact on microbial community structure [15]. Studies have also shown that secretions from plant roots can recruit specific microorganisms to establish unique communities, resulting in significant differences in rhizosphere communities across cultivars or genotypes [16].

*Paeonia lactiflora* Pall, a perennial herb of the family Paeoniaceae and native to temperate Eurasia, is a well-known traditional Chinese species with ornamental and medicinal value [17,18,19]. As the species is extremely diverse, with more than 2200 new cultivars generated through breeding programs between 1900 and 2020 [20], it is important to explore the composition of the rhizosphere microbial community and tap the microbial resources for sustainable and healthy development of the *P. lactiflora* industry. This can be achieved using high-throughput sequencing, which has made deciphering the rhizosphere microbiome of any species increasingly accurate and efficient owing to rapid development and not relying on cumbersome plate-culture methods [21]. 

Nowadays, microbial products have unlimited potential as biofertilizers, biopesticides and biostimulators, and they are considered green and sustainable [22]. Microbial inoculants play an important role in improving crop production and adapting to changing environmental condition [23]. Understanding the communication between plants and rhizosphere microbes can help identify ecologically friendly strategies that can inhibit plant diseases and improve plant growth and yield [24]. For example, through the investigation of the rhizosphere bacterial community of the crop *Amaranthus* spp., the bacterial groups that are important for its adaptation to various habitat conditions have been identified [25]. Rhizosphere microbes also play an important role in plant–insect interactions in maintaining crop health [26]. *P. lactiflora* is a famous ornamental plant, its production and cultivation are restricted by a variety of factors, such as high temperature, drought, pests and diseases [27,28,29]. Therefore, it is important to explore the relationship between *P. lactiflora* cultivars and rhizosphere microbial community, and to develop beneficial bacteria which can help the growth and development of *P. lactiflora* and have application potential in production and cultivation. 

In previous studies, we analyzed the rhizosphere microbiomes of, and soil factors affecting, four ornamental cultivars of *P. lactiflora*, and studied the influence of different soil factors on the composition of rhizosphere microbiomes of these cultivars [30]. However, the effects of different cultivars of *P. lactiflora* on their rhizosphere microbiomes remain to be explored. Therefore, in this study, we analyzed the rhizosphere bacterial communities of eight common cultivars of *P. lactiflora* using high-throughput sequencing technology and compared the differences between their composition. The findings of this study will lay the foundation for understanding the influence of different cultivars of *P. lactiflora* on their rhizosphere microbial community structures and to explore and tap rhizosphere microbial resources.

## 2. Materials and Methods

### 2.1. Plant Material and Sample Collection 

The study was conducted in the germplasm repository of the College of Horticulture and Landscape Architecture, Yangzhou University, Jiangsu Province, China (32°30′ N, 119°25′ E). The region exhibits a subtropical monsoon climate, with an average annual precipitation of 991 mm and an average annual temperature of 15.2 °C. Plants were grown on the land *P. lactiflora* where has been growing since 2008. Apart from watering and weeding, no other treatment was performed since planting to minimize any spatial changes in physical and chemical properties. At the end of October 2022, we selected eight cultivars of *P. lactiflora*, namely “Bing Shan” (BS), “Duo Ye Zi” (DYZ), “Lian Tai” (LT), “Sheng Tao Hua” (STH), “Tao Hua Fei Xue” (THFX), “Xue Feng” (XF), “Zi Hong Zheng Hui” (ZHZH) and “Zi Lou Xian Jin” (ZLXJ), for analyses. Their age and physiological status are consistent, which is conducive to removing the influence of growth years and health status on the data. We selected three plants per cultivar, with each plant more than 5 m apart from the other. We carefully dug out the roots and shook off the clumps and particles of soil. Then, we used a sterile brush to carefully remove the soil attached to the roots, which was the rhizosphere soil. Next, plant debris was removed from the rhizosphere soil, which was then placed in a sterile centrifuge tube and immediately frozen using liquid nitrogen. It was thereafter stored at −20 °C until microbiome analysis.

### 2.2. DNA Extraction, Amplification and Sequencing

Genomic DNA from rhizosphere soil samples was extracted using the cetyltrimethylammonium bromide method [31]. Thereafter, we evaluated the DNA content and purity using 1% agarose gel electrophoresis. DNA was then diluted to 1 ng·μL^−1^ using sterile water. We amplified the V4 region of the 16S rRNA gene in each sample using the primers 515F (5′-GTGCCAGCMGCCGCGGGG-3′) and 806R (5′-GGACTACHVGGGTWTCTAAT-3′) to generate bacterial libraries. These primers have a unique six-nucleotide-long barcode at the 5′ end of the forward primer. Subsequently, we performed polymerase chain reaction (PCR) using the Phusion^®^ High-Fidelity PCR Master Mix (New England Biolabs, Ipswich, MA, USA). Then, we mixed the PCR products with an equal volume of 1× loading buffer (containing SYB green) and subjected them to 2% agarose gel electrophoresis. The PCR products were purified using a GeneJETTM Gel Extraction Kit (Thermo Scientific, Waltham, MA, USA). After that, sequencing libraries were generated, and index codes were added using a Truseq^®^ DNA PCR-Free Sample Preparation Kit (Illumina, San Diego, CA, USA). We assessed library quality using a Qubit^@^ 2.0 Fluorometer (Thermo Scientific) and the Agilent Bioanalyzer 2100 system. At last, the libraries were sequenced using the Illumina NovaSeq 6000 platform, and paired reads were generated.

### 2.3. Sequence Analysis

All raw data were analyzed using the SMRT Link analysis software (version 9.0; https://www.pacb.com/wp-content/uploads/SMRT_Link_Installation_v90.pdf, accessed on 11 May 2023). Cyclic consistent sequencing reads were obtained, and raw reads were processed to filter the sequences for length (<800 or >2500 bp) and quality. After quality control, all high-quality reads were selected using UNOISE2 [32] with default parameters in USEARCH v10 software [33] and assigned to amplicon sequence variants (ASVs). The representative sequences were then classified using the BLAST algorithm of the SILVA reference database (version 12.8) in QIIME 1.91 [34].

### 2.4. Biomarkers Analysis of Cultivars

Linear discriminant analysis (LDA) effect size (LEfSe) analysis was performed to identify the biomarkers of the cultivars. R package “microbiomrMarker” (version 1.0.1) was used for the analysis and visualization. Non-parametric Kruskal–Wallis rank sum test was used to detect bacteria with significant abundance difference among cultivars. Then, for the significantly different bacteria obtained in the previous step, the group Wilcoxon rank sum test was used to analyze the difference between cultivars. Finally, linear discriminant analysis (LDA) was used to reduce the data and assess the impact of significantly different species (LDA score).

### 2.5. Analysis of Differential Bacterial ASVs with Different Abundances

We performed differential analysis on the ASVs using DESeq2 with negative binomial generalized linear models [35]. Genera with a *p* value < 0.05 and |log2 (fold change)| > 1 were regarded as significantly different. We further used the R packages “ggrepel” (version 0.9.1) “ggplot2” (version 2.15.3) and “tidyverse” (version 1.3.1) to visualize the data [36,37,38].

### 2.6. Co-Occurrence Network Analysis

We also performed co-occurrence network analysis for the rhizospheres of different cultivars. Firstly, the data were preprocessed, and ASVs with a relative abundance < 0.2% were removed. Then, the microbial co-occurrence network index was calculated using the R package “WGCNA” (version 4.1.2); [39], and Gephi (version 0.9.2) was used to visualize the co-occurrence network [40].

### 2.7. Statistical Analysis

To analyze α diversity, Pielou’s evenness, richness and Shannon’s index were calculated using QIIME (version 1.7.0) and presented using the R package “ggplot2” (version 2.15.3); [36]. To examine the difference between samples, β diversity analysis was performed using QIIME (version 1.9.1). 

## 3. Result

### 3.1. The Abundance and Diversity of Rhizosphere Bacteria Associated with Different Cultivars of P. lactiflora 

**Figure 1:**
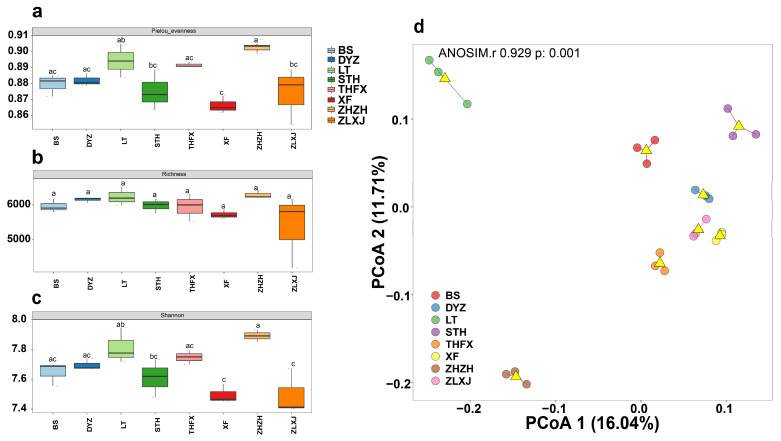
α and β diversity analyses of rhizosphere microorganisms associated with different cultivars of *P. lactiflora*. (**a**) Pielou’s evenness of bacterial communities in different cultivars. (**b**) The richness of bacterial communities in different cultivars. (**c**) Shannon’s diversity index of bacterial communities in different cultivars. (**d**) Principal coordinate analysis (PCoA) of bacterial communities. Values with different letters are significantly different at *p* < 0.05. The triangle represents the center point of the samples in each group.

To compare the diversity and evenness of rhizosphere bacteria associated with different *P. lactiflora* cultivars, Pielou’s evenness, richness and Shannon’s index were calculated. The results showed that Pielou’s evenness was the highest for ZHZH and the lowest for XF (Figure 1a). This indicated that the evenness of rhizosphere bacteria was the highest and lowest in ZHZH and XF, respectively. There was no significant change in the richness index of the rhizosphere microorganisms of the eight cultivars, which means there was no significant difference in the richness of rhizosphere bacteria among the eight cultivars (Figure 1b). Similarly, Shannon’s index was the highest for ZHZH and the lowest for XF and ZLXJ, and there was no significant difference among BS, DYZ, LT THFX and ZHZH, suggesting that ZHZH exhibited the highest rhizosphere bacterial diversity, whereas XF and ZLXJ exhibited the lowest rhizosphere bacterial diversity (Figure 1c). Additionally, α diversity analysis revealed that there were considerable differences in rhizosphere bacterial diversity among the eight cultivars.

In addition, based on ASV levels, we performed principal coordinate analysis (PCoA) to determine the differences between samples. We noted that the contribution values of PCoA1 and PCoA2 to the differences in rhizosphere bacteria associated with eight *P. lactiflora* cultivars were 16.04% and 11.71%, respectively (Figure 1d). Figure 1d indicates that the scattered points corresponding to the samples in the group are clustered and show good repeatability. The groups are far from each other, and the degree of differentiation is good.

### 3.2. Composition and Abundance of Rhizosphere Bacterial Communities Associated with Different Cultivars of P. lactiflora

A total of 11,004 ASVs were identified, including 21 phyla, 51 classes, 60 orders, 142 families, 397 genera and 563 species. Among them, Actinobacteria, Proteobacteria, Acidobacteria, Bacteroidetes, Firmicutes, Verrucomicrobia, Planctomycetes and Chloroflexi were the dominant phyla (relative abundance > 1%), accounting for 36.9%, 31.3%, 12.3%, 2.6%, 2.2%, 2.0%, 1.2% and 1.1%, respectively. Of these, Actinobacteria, Proteobacteria and Acidobacteria were significantly more abundant than any other phyla and were, therefore, the primary phyla in the samples.

**Figure 2:**
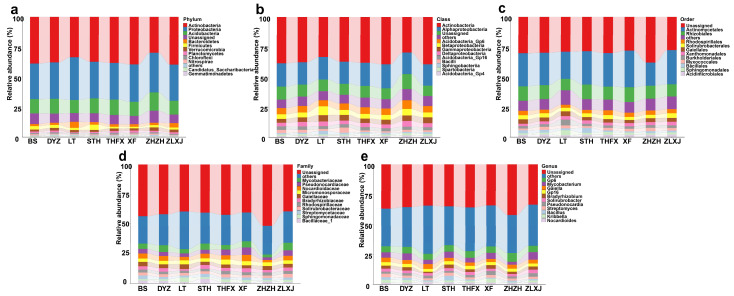
Composition of rhizosphere bacterial communities associated with different cultivars. Relative abundance of bacterial phylum (**a**), class (**b**), order (**c**), family (**d**) and genus (**e**).

By analyzing and comparing the distribution of rhizosphere bacteria in different classification levels, we found that the bacterial compositions associated with the cultivars of *P. lactiflora* were highly similar at all taxonomic levels, but their relative abundance varied. At the phylum level, the relative abundance of Actinobacteria, Proteobacteria and Acidobacteria in the rhizospheres of the cultivars was much higher than that of other phyla. Furthermore, the relative abundance of Firmicutes in STH was higher than that of other cultivars, and the relative abundance of Bacteroidetes in LT was higher than that in others (Figure 2a). At the class level, Betaproteobacteria and Sphingobacteria were relatively abundant in LT, whereas at the order level, the relative abundance of the dominant bacteria Actinomycetales was significantly lower in ZHZH compared to that in other cultivars (Figure 2b). At the genus level, the relative abundance of *Kribbella* was higher in XF and lower in LT and ZHZH (Figure 2e).

### 3.3. Identification of Biomarker Taxa in the Rhizosphere of Different Cultivars of P. lactiflora

**Figure 3:**
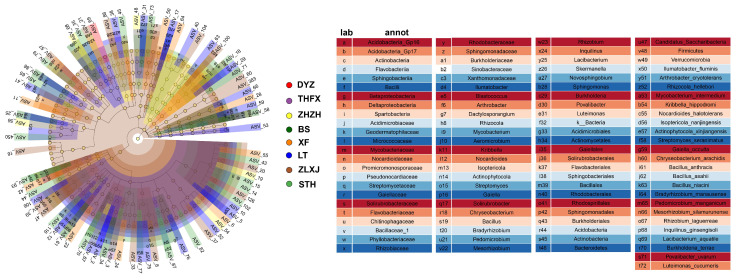
Linear discriminant analysis (LDA) effect size (LEfSe) of the rhizosphere bacterial communities associated with different cultivars of *P. lactiflora*. The cladogram indicates the phylogenetic distribution of microbial lineages associated with the plant compartments. Circles represent phylogenetic levels from kingdom to genus.

Linear discriminant analysis (LDA) effect size (LEfSe) analysis identified 232 bacterial biomarkers with significantly different abundance in the samples (Figure 3). The bacterial biomarkers included 7 phyla, 11 classes, 18 orders, 29 families, 41 genera and 51 species. Approximately 23, 2, 63, 41, 4, 30, 17 and 52 biomarkers were identified to be associated with BS, DYZ, LT, STH, THFX, XF, ZHZH and ZLXJ, respectively.

We noted that STH and ZLXJ exhibited the maximum number of biomarkers. STH exhibited biomarkers from two phyla (Acidobacteria and Firmicutes), three classes (Acidobacteria_Gp16, Acidobacteria_Gp17 and Bacilli), one order (Bacillales), three families (Micrococcaceae, Bacillaceae_1 and Xanthomonadaceae) and three genera (*Arthrobacter*, *Bacillus* and *Luteimonas*), whereas ZLXJ exhibited biomarkers from two phyla (Actinobacteria and Verrucomicrobia), three classes (Actinobacteria, Betaproteobacteria and Spartobacteria), four orders (Acidimicrobiales, Actinomycetales, Solirubrobacterales and Burkholderiales), five families (Acidimicrobiaceae, Mycobacteriaceae, Pseudonocardiaceae, Solirubrobacteraceae and Burkholderiaceae) and six genera (*Ilumatobacter*, *Mycobacterium*, *Solirubrobacter*, *Bradyrhizobium*, *Skermanella* and *Burkholderia*). In contrast, DYZ and THFX exhibited the least number of biomarkers. Although ZHZH has the highest diversity, it does not show significant differences in abundance among the cultivars BS, DYZ, LT and THFX, so LEfSe analysis did not produce the largest number of biomarkers.

### 3.4. Differences in the Abundance of ASVs in Different Cultivars of Rhizosphere Bacteria 

**Figure 4:**
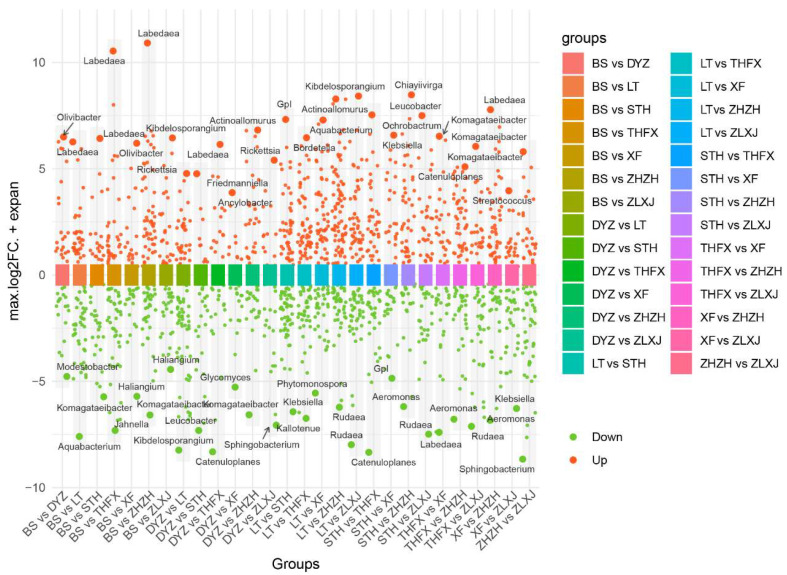
Volcano plots depicting enriched and depleted bacteria in the rhizosphere associated with each cultivar comparison.

DESeq2 was used to compare the rhizosphere bacteria associated with the eight cultivars and to detect differential bacterial ASVs with different abundances, it was visualized by differential analysis volcano map (Figure 4). The results showed that multiple ASVs with different abundance were found in different comparison groups. LT vs. ZHZH exhibited the maximum number of differential ASVs (116), of which 74 ASVs were enriched and 42 ASVs were depleted. Among them, *Rudaea* was significantly enriched in LT, and *Actinoallomurus* was significantly enriched in ZHZH. In addition, LT vs. STH and LT vs. THFX exhibited a high number of differential ASVs (97). In LT vs. STH, *Klebsiella* was significantly enriched in LT, whereas in LT vs. THFX, *Kallotenue* was significantly enriched in LT and *Bordetella* in THFX. DYZ vs. XF exhibited the least number of differential ASVs (only 24), of which 9 ASVs were enriched and 15 were depleted. Furthermore, *Glycomyces* was significantly enriched in DYZ and *Ancylobacter* in XF. In general, among the eight cultivars, ASV abundance was the most variable between LT and ZHZH, as well as between STH and THFX, and the most similar between DYZ and XF.

### 3.5. Co-Occurrence Network Analysis of Rhizosphere Bacteria Associated with Different Cultivars of P. lactiflora 

**Figure 5:**
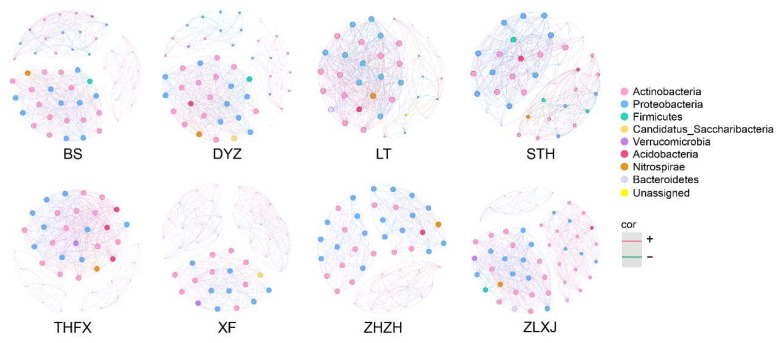
The co-correlation networks of bacterial ASVs associated with different cultivars of *P. lactiflora*. The size of each node is proportional to the number of connections (i.e., degree), and the nodes are colored according to phyla. Red and green edges indicate positive and negative correlations, respectively.

We screened bacterial ASVs with abundance > 0.2% associated with the eight cultivars and constructed rhizosphere bacterial interaction network diagrams (Figure 5). On average, there were 45 nodes and 378 edges in each co-occurrence network diagram. Among them, LT exhibited the fewest nodes, and STH exhibited the fewest edges, while ZLXJ exhibited the maximum number of nodes and edges. In other cultivars, except LT (49.7%), the proportion of positive correlation edges was >50%. In THFX, there were 393 positively correlated edges (98.3%) and seven negatively correlated edges (1.7%). Except for LT (21.9%) and THXF (22.2%), the modularity indexes of the networks in other cultivars were >40%, which means they exhibited strong modular structures. Except for DYZ, which exhibited four main modules, the rest of the networks exhibited three main modules. The nodes in the network were divided according to phylum, and in the main modules of all cultivars, nodes with the largest number belonged to Actinobacteria and Proteobacteria, whereas other phyla were relatively rare.

## 4. Discussion

As the soil is directly affected by root deposition, the rhizosphere has the most complex, diverse and active microbiota [41]. These microorganisms play vital roles in plant growth and development; for example, the symbiosis between plants and microorganisms makes plants better adapted in terms of growth, nutrient cycling, pathogen resistance, and stress resistance [42]. Of them, bacteria are the most abundant group of rhizosphere microorganisms [43]. Therefore, in this study, we studied the rhizosphere bacterial communities of eight cultivars of *P. lactiflora* using high-throughput sequencing technology to explore the differences and similarities between them.

Differences in the rhizosphere microbiome of different cultivars of the same species result in changes in plant adaptability. In the study by Claudia et al., cultivars affected the rhizosphere microbial community structure and functional adaptation to drought in wheat [44]. The rhizosphere microbiome structure of different cultivars can also show plant resistance to diseases to some extent [45]. In the study by Dubey et al., resistant cultivars of soybean exhibited more beneficial flora than the sensitive cultivars [46]. Similarly, while studying *Verticillium* in cotton, it was found that the resistant cultivars exhibited a higher abundance of beneficial microbiota, such as *Bacillales*, *Pseudomonadales*, *Rhizobiales* and *Trichoderma*, than the sensitive cultivars [47]. In this study, STH and BS exhibited a higher abundance of *Bacillales* and *Rhizobiales*, and thus, we hypothesized that STH and BS may exhibit increased resistance to diseases compared to other cultivars. 

The results of this study showed that Actinobacteria, Proteobacteria, Acidobacteria, Bacteroidetes, Firmicutes, Verrucomicrobia, Planctomycetes and Chloroflexi were the dominant phyla in the microbiomes of *P. lactiflora* cultivars, among which the relative abundance of Actinobacteria, Proteobacteria and Acidobacteria was much higher than that of other phyla. This finding has also been confirmed in *Cathaya argyrophylla* and Chinese fir [48,49]. We also noted that the rhizosphere bacterial communities associated with *P. lactiflora* exhibited high diversity and abundance. 

According to the literature, Actinobacteria can be used as biofertilizers, providing nutrients to plants, promoting growth, alleviating biotic and abiotic stresses, and enhancing resistance to pathogens [50]. *Proteobacteria* can respond to heavy metal stress and have the potential for biological remediation [51]. In addition, they play an important role in nitrogen recycling, which can promote plant growth, increase yield, and improve fruit and seed quality [52].

In this study, α and β diversity analyses revealed that the rhizosphere bacterial composition of the eight cultivars was similar to some extent, but the relative abundance of different bacterial groups was different at different taxonomic levels. At the phylum level, STH exhibited the highest abundance of *Firmicutes*, which have sulfate- and iron-reduction abilities and play an important role in bioremediation [53]. In addition, they play a central role in lignocellulosic decomposition and hemicellulose degradation [54]. In *Vallisneria spiralis*, the genus *Bacillus* in phylum Firmicutes promotes plant growth by biofixing nitrogen to produce IAA and GA [55]. The relative abundance of Bacteroidetes was higher in LT than in other cultivars. *Bacteroidetes* exist widely in the soil ecosystem and are sensitive biological indicators of agricultural soil [56,57]. *Bacteroidetes* can also regulate the functions of the carbon cycle and microbial community, and are closely related to the degradation of organic matter [58,59,60]. When using plant growth promoting rhizobacteria to study the growth of maize, it was found that flavobacterium belonging to Bacteroidetes had the ability to promote the growth and/or antagonize soil-borne fungal diseases [61]. Therefore, STH and LT may be rich in microbial resources related to bioremediation and organic degradation.

Compared to STH, *Klebsiella* was significantly enriched in LT. *Klebsiella* plays a variety of roles. A study found that *Klebsiella pneumoniae* 342 helped fix nitrogen to reduce nitrogen deficiency in wheat [62]. *Klebsiella* sp. D5A promoted plant growth and increased salt tolerance [63]. *Klebsiella variicola* SURYA6 has been shown to produce large amounts of plant-growth-promoting, salt-ameliorating and antioxidant metabolites, and it is a potential biological inoculant for salt stress management in plants [64]. We thus hypothesized that LT exhibited salt-resistant potential and a higher nitrogen fixation capacity than STH. *Ancylobacter*, which was significantly enriched in XF, has been shown to participate in environmental remediation, such as the utilization of dichloromethane, methanol, formate and formaldehyde, and the oxidation of arsenite in contaminated water and groundwater [65,66]. *Ancylobacter pratisalsi* sp isolated from the rhizosphere of *Plantago winteri* was found to promote plant growth [67]. It can be suggested that XF may have the potential for soil remediation and plant growth. 

The rhizosphere microbial community is not only a collection of independent individuals but also an interrelated ecological community complex, which can communicate, cross-feed, recombine, and coevolve with time [68]. Co-occurrence network analysis emphasizes the understanding of microbiome co-occurrence characteristics and interaction patterns in ecosystems from a network perspective [69]. In the rhizosphere bacterial co-occurrence network analysis of different cultivars, the number of positively correlated edges was more than that of negative edges in all cultivars, except in LT. This indicated that in LT, rhizosphere bacteria exhibited more competitive and predatory relationships. However, in other cultivars, especially in THFX, rhizosphere bacteria survived mostly through cooperation. It was further noted that the rhizosphere bacterial communities associated with different cultivars of *P. lactiflora* exhibited different survival strategies to adapt to the root microclimate and achieve stability. In addition, in the co-occurrence network analysis, the key nodes were mainly obtained from Actinobacteria and Proteobacteria, exhibiting high abundance. This suggested that highly abundant bacteria played a more important role in maintaining the stability of the rhizosphere bacterial community.

## 5. Conclusions

This study carried out microbiome analysis to compare the composition of rhizosphere bacterial communities associated with eight *P. lactiflora* cultivars. Results showed that Actinobacteria, Proteobacteria, Acidobacteria, Bacteroidetes, Firmicutes, Verrucomicrobia, Planctomycetes and Chloroflexi were the dominant phyla. The composition of the rhizosphere bacterial community of eight cultivars were highly similar, but slightly differences in relative abundance. ASV abundance was most different between LT and ZHZH, STH and THFX but similar between DYZ and XF. There were most biomarkers in STH and ZLXJ. In most cultivars, rhizosphere bacteria maintain homeostasis by cooperation, and Actinobacteria and Proteobacteria play an important role. STH and LT were rich in microbial resources related to bioremediation and organic degradation, whereas STH and BS were rich in resources exhibiting higher resistance to diseases.

## Data Availability

Not applicable.
